# Utilizing maternal healthcare services: are female-headed households faring poorly?

**DOI:** 10.1186/s12884-024-06445-8

**Published:** 2024-04-22

**Authors:** Subhasree Ghatak, Meghna Dutta

**Affiliations:** 1 Research Scholar, Department of Humanities and Social Sciences, Indian Institute of Technology Patna, Patna, India; 2 Assistant Professor of Economics, Department of Humanities and Social Sciences, Indian Institute of Technology Patna, Patna, India

**Keywords:** Maternal healthcare, Antenatal care, Skilled birth assistance, Postnatal care, Sex of household head, India

## Abstract

**Background:**

Utilization of maternal healthcare services has a direct bearing on maternal mortality but is contingent on a wide range of socioeconomic factors, including the sex of the household head. This paper studies the role of the sex of the household head in the utilization of maternal healthcare services in India using data from the National Family Health Survey-V (2019–2021).

**Methods:**

The outcome variable of this study is maternal healthcare service utilization. To that end, we consider three types of maternal healthcare services: antenatal care, skilled birth assistance, and postnatal care to measure the utilization of maternal healthcare service utilization. The explanatory variable is the sex of the household head and we control for specific characteristics at the individual level, household-head level, household level and spouse level. We then incorporate a bivariate logistic regression on the variables of interest.

**Results:**

24.25% of women from male-headed households have complete utilization of maternal healthcare services while this proportion for women from female-headed households stands at 22.39%. The results from the bivariate logistic regression confirm the significant impact that the sex of the household head has on the utilization of maternal healthcare services in India. It is observed that women from female-headed households in India are 19% (AOR, 0.81; 95% CI: 0.63,1.03) less likely to utilize these services than those from male-headed households. Moreover with higher levels of education, there is a 25% (AOR, 1.25; 95% CI: 1.08,1.44) greater likelihood of utilizing maternal healthcare services. Residence in urban areas, improved wealth quintiles and access to healthcare facilities significantly increases the chances of maternal healthcare utilization. The interaction term between the sex of the household head and the wealth quintile the household belongs to, (AOR, 1.39; 95% CI: 1.02, 1.89) shows that the utilization of maternal healthcare services improves when the wealth quintile of the household improves.

**Conclusion:**

The results throw light on the fact that the added expenditure on maternal healthcare services exacerbates the existing financial burden for the economically vulnerable female-headed households. This necessitates the concentration of research and policy attention to alleviate these households from the sexual and reproductive health distresses.

**Trial Registration:**

Not Applicable.

**JEL Classification:**

D10, I12, J16.

## Introduction

Despite significant advancements in medical science and technology, maternal mortality remains a global issue, particularly in underdeveloped countries. Maternal death is defined as “the death of a woman while pregnant or within 42 days of termination of pregnancy, irrespective of the duration and site of pregnancy, from any cause related to or aggravated by the pregnancy or its management but not from accidental or incidental causes” [1].Estimates from the World Health Organization state that low and lower-middle-income countries account for 94% of maternal deaths worldwide, and women in low-income countries are at 130 times higher risk of maternal mortality than women in high-income countries [1]. The concern over maternal health i.e. health of the woman pertaining to her pregnancy, childbirth and the post-partum period, resulted in the formulation of Sustainable Development Goals (henceforth, SDG) by the United Nations in 2015, which considered maternal health to be of utmost importance. The SDG aims to reduce the global Maternal Mortality Ratio (henceforth, MMR), defined as the number of maternal deaths during a given time period per 100,000 live births during the same time period to less than 70 maternal deaths per 100,000 live births by 2030 [1]. The achievement of this target requires a transitional shift from high maternal mortality to low maternal mortality all over the world. Among the countries known for high MMR, India accounted for one-fifth of the global maternal deaths in 2015 [2]. Obstetric transition is divided into five stages. The first stage is when MMR is greater than 1000, followed by the second stage where MMR ranges between 999 − 300. The third stage is when MMR is between 299 − 50, the fourth stage is characterized by an MMR less than 50 and lastly the fifth stage where avoidable deaths are zero. India is characteristic of the third stage in obstetric transition, i.e., MMR between 299 − 50 maternal deaths per 100,000 live births. A special bulletin in India by the Sample Registration System on Maternal Mortality states that MMR in India stands at 103 per 100,000 live births for the period 2017-19. Though a decline from 113 for the period 2016-18, the country is yet to achieve its set target of reducing MMR to less than 70 deaths per 100,000 live births. The geographical vastness and socio-cultural diversity result in significant interstate and intra-state variations in maternal mortality, making uniform implementation of health-sector reforms difficult. This implies an urgent need for a systematic study to identify the structural and proximal factors responsible for reducing the maternal mortality rate and formulating policies based on the findings.

Utilization of maternal healthcare services, which includes Antenatal Care (henceforth, ANC), Skilled Birth Assistance (henceforth, SBA), and Postnatal Care (henceforth, PNC) during pregnancy, childbirth, and post-delivery significantly reduces maternal mortality [3–7]. The utilization of these services is contingent on a whole host of factors, viz. socioeconomic conditions, availability and accessibility of healthcare services, knowledge of pregnancy-related appropriate health behaviours, maternal age, educational attainment, exposure to mass media, residential area, and decision-making capacity [6–10]. The decision to seek healthcare, especially maternal healthcare, generally involves interactions among the women, their partners, and family members [7]. Healthcare seeking, therefore, is heavily influenced by household dynamics. However, what remains ubiquitously absent in the existing literature is a study of the gender aspect of maternal healthcare seeking. Since for a woman her socioeconomic context largely determines her maternal healthcare utilization, as discussed above, it becomes imperative to divert research attention to study the influence of household headship on such utilization. Of the few studies that have interrogated the role of household headship on maternal healthcare utilization, a study in West Bengal, India, found that female headship reduces the chances of availing of ANC services [11]. On the other hand, studies in Ethiopia, Gabon, Indonesia, and Sub-Saharan Africa reveal that the odds of maternal healthcare utilization increase with a female head [7, 12–14]. Women in female-headed households were more likely to use facility-based delivery than women from male-headed households [9]. The share of expenditure on maternal healthcare services is greater in female-headed households because of greater autonomy and decision-making capacity than their male-headed counterparts [15]. As women moved up to higher age brackets, the odds of them availing of maternal healthcare services increased [6, 7, 13, 16]. Previous studies also confirm that for women with higher levels of educational attainment, the chances of utilizing maternal healthcare services are significantly improved [8, 13, 17, 18]. Studies in different country contexts provide evidence that a woman’s parity or birth order is an essential factor determining the utilization of maternal healthcare services for the current birth. An inverse relationship has been established between parity and maternal healthcare utilization [13, 17–19]. Evidence from different study settings shows that women from wealthier households or women employed in paid activity were more likely to utilize healthcare services [10, 14, 20].

Our study adds to the literature on maternal health by explicitly studying the relationship between the sex of the household head and different components of maternal healthcare, i.e., ANC, SBA, and PNC. The lack of evidence in the Indian context that can postulate the nature of the relationship between the sex of the household head and the utilization of various maternal healthcare services stands as the primary motivation behind this study. With the increasing scholarship on the gendered aspects of household headship, this study will be a timely intervention to assess its impact on maternal healthcare service utilization, thereby impacting maternal mortality.

The rest of the paper is organized as follows: Sect. 2 deals with the data and study variables. Section 3 states the model specification and empirical analysis. Section 4 presents the results, followed by a robustness check in Sect. 5. Section 6 discusses the findings, and Sect. 7 concludes the study.

## Data and study variables

### Data source

This paper uses secondary data from the National Family Health Survey-V (henceforth, NFHS). It is a nationally representative, large-scale household survey conducted by the Ministry of Health and Family Welfare, Government of India. The present set of data pertains to 2019–2021. The total sample of NFHS-5 comprises 724,115 women and 101,839 men. The women interviewed were in the age bracket of 15–49 years, which corresponds to the reproductive age for women. However, for our study, we have considered women who were ever married and had at least one live birth in the last five years prior to the survey. This reduces the sample to 176,601 women. Accounting for the availability of data on the covariates, which would be required to explain the relationship, the study considers 26,944 women.

### Outcome variable

The outcome variable of this study is maternal healthcare service utilization. (*mhcu*). We employ three categories of maternal healthcare service utilization, i.e., ANC, SBA, and PNC, described below.

1. ANC: We use four indicators to create this variable. The number of ANC visits should be at least 4; the first antenatal visit should take place in the first trimester, consumption of iron folic tablets and syrups for at least 100 days, and at least two tetanus toxoid injections should be taken before birth. If all of the above is answered affirmatively, it is coded as “1,” indicating ANC, and “0” otherwise.
2. SBA: If the delivery has been assisted by a doctor, auxiliary nurse midwifery, nurse or midwife, or a health visitor or has taken place in a government or municipal hospital, dispensary, rural hospital, private hospital, maternity home, and the like, it has been coded as “1” and if the delivery has been only assisted by a traditional birth assistant, ‘dai,’ friend or relative or has taken place at the respondent’s home or the parent’s home, it has been coded as “0”.
3. PNC: We consider four indicators for this variable, i.e., whether or not the respondent’s health was checked before and after discharge/ delivery at home and whether or not the healthcare provider counseled on the danger signs for newborns and on breastfeeding. Having answered in affirmation in at least two of the four is coded as “1,” indicating PNC, and “0” otherwise.

If the respondent answered affirming all three categories, it has been considered as full utilization of maternal healthcare services. If the respondent answered in negation to any of the three categories, it has been considered an incomplete utilization of maternal healthcare services. Complete utilization is coded as ‘1’ and incomplete as ‘0’.

### Description of explanatory variables

The variable of interest and other control variables are discussed at length in Table 1.

**Table 1:** Description of explanatory variables

Variable	Nature	Description and coding
*Headship characteristics*		
1. Sex of the household head (sexhhd)	Categorical	Headship, as reported in the survey. 0: male, 1: female
2. Relationship to the household head	Categorical	1: Head herself, 2: Spouse, 3: Daughter, 4: Daughter-in-law, 5: Others
*Individual characteristics*		
3. Maternal age	Continuous	Age of the woman at the time of the survey
4. Woman’s educational attainment	Categorical	0: formal education, 1: primary education, 2: secondary education, 4: higher education
5. Parity	Continuous	Number of living children of the respondent.
6. Working Status of the respondent	Continuous	1: Respondent was working at the time of the survey, 0: Otherwise
7. Health Insurance coverage	Categorical	1: Covered by health insurance, 0: Otherwise
8. Possession of bank account	Categorical	1: Possession of an account in a bank or any other financial institution by the woman, 0: Otherwise
9. Exposure to mass media	Categorical	Measured using three indicators: Frequency of reading the newspaper/magazine, frequency of listening to the radio, and frequency of watching television. 1: Exposure to any two out of the three metrics, 0: Otherwise
10. Woman’s Participation Index	Categorical	Created out of three decision-making items in the household- the decision to spend on the respondent’s healthcare, the decision to spend on large household purchases, and the decision to visit relatives and friends. 1: Sole decision taker or takes the decision jointly with her spouse, 0: Otherwise
11. Sex of the last child	Categorical	1: Male, 2: Female
*Household characteristics*		
12. Religion	Categorical	0: No Religion, 1: Hindu, 2: Muslim, 3: Christian, 4: Sikh, 5: other minority religion
13. Wealth Index	Categorical	1: Poorest, 2: Poorer, 3: Middle, 4: Richer, 5: Richest
14. Residence	Categorical	0: Urban, 1: Rural
*Spouse Characteristics*		
15. Partner’s educational attainment	Categorical	0: No education, 1: Primary, 2: Secondary, 3: Higher
16. Partner’s age	Continuous	Age of the spouse at the time of the survey
*Healthcare Provision*		
17. Type of facility	Categorical	0: Other, 1: Public facility, 2: Private facility

*Source* Authors’ calculations

### Sample description

Table 2 presents the descriptive statistics of the variables. Of the 26,944 women in the study sample, 84.39%, i.e., 22,737 women, belonged to male-headed households, and the rest, 15.61%, i.e., 4207 women, belonged to female-headed households. 24.25% and 22.39% of women from male-headed and female-headed households, respectively, have complete utilization of maternal healthcare services. Table 3 exhibits the percentage of women utilizing each component of maternal healthcare service across household headships. Women from female-headed households have lower utilization of all three components, i.e., ANC, SBA, and PNC.

**Table 2:** Descriptive statistics

*Variables*	Observations		%		Mean	Std. Dev.
**Male-headed households (MHH)**	22737		84.39			
**Female-headed households (FHH)**	4207		15.61			
**Age**	26944				27.5	5.2
**Educational attainment**	MHH	FHH	MHH	FHH		
No education	4519	959	19.88	22.80		
Primary	2848	520	12.53	12.36		
Secondary	11947	2121	52.54	50.42		
Higher	3423	607	15.05	14.43		
**Parity**	26944				2.12	1.26
**Working Status**	MHH	FHH	MHH	FHH		
Working	4492	874	19.76	20.77		
Not working	18245	3333	80.24	79.23		
**Health Insurance coverage**	MHH	FHH	MHH	FHH		
Yes	6252	1258	27.50	29.90		
No	16485	2949	72.50	70.10		
**Exposure to mass media**	MHH	FHH	MHH	FHH		
Yes	6914	1254	30.41	29.81		
No	15823	2953	69.59	70.19		
**Woman’s Participation Index**	MHH	FHH	MHH	FHH		
Yes	18137	3533	79.77	83.98		
No	4600	674	20.23	16.02		
**Possession of bank account**	MHH	FHH	MHH	FHH		
Yes	18061	3458	79.43	82.20		
No	4676	749	20.57	17.80		
**Sex of the last child born**						
Male	14,377		53.36			
Female	12,527		46.64			
**Partner’s educational attainment**	MHH	FHH	MHH	FHH		
No education	3075	764	13.52	18.16		
Primary	2871	500	12.63	11.88		
Secondary	12789	2287	56.25	54.36		
Higher	3918	630	17.23	14.98		
**Partner’s age**	26944				31.88	6.38
**Religion**	MHH	FHH	MHH	FHH		
Hindu	16710	2905	73.49	69.05		
Muslim	3319	601	14.60	14.29		
Christian	1669	503	7.34	11.96		
Sikh	446	76	1.96	1.81		
Others	593	122	2.61	2.90		
**Residence**	MHH	FHH	MHH	FHH		
Rural	17831	3323	78.42	78.99		
Urban	4906	884	21.58	21.01		
**Wealth quintile**	MHH	FHH	MHH	FHH		
Poorest	5603	1184	24.64	28.14		
Poorer	5199	969	22.87	23.03		
Middle	4464	851	19.63	20.23		
Richer	4107	643	18.06	15.28		
Richest	3364	560	14.80	13.31		

*Source* Authors calculations

*Note* Here, MHH represents Male-Headed Households, and FHH represents Female-Headed Households

**Table 3:** Percentage of women utilizing each component of maternal healthcare service across household headship

Components of Maternal Healthcare Service	Male-Headed Households	Female-Headed Households
ANC	27.37	25.89
SBA	91.32	88.78
PNC	87.79	86.52

*Source* Authors’ calculations

Furthermore, we provide in Table 4, the percentage of women having complete utilization of maternal healthcare services in both male-headed and female-headed households by all the categories of the independent variables used in the study.

**Table 4:** Percentage of women having complete utilization of maternal healthcare services by the categories of independent variables

	complete maternal healthcare utilization (in %)		
Independent variables	male-headed households	female-headed households	
Educational attainment			
No education	14.34	11.68	
Primary	17.84	14.04	
Secondary	26.12	24.85	
Higher	36.11	37.89	
**Working Status**			
Working	24.58	23.91	
Not working	24.17	21.99	
**Health Insurance coverage**			
Yes	27.4	26.07	
No	23.05	20.82	
**Exposure to mass media**			
Yes	31.82	30.7	
No	20.94	18.86	
**Woman’s Participation Index**			
Yes	25.26	22.79	
No	20.26	20.33	
**Possession of bank account**			
Yes	25.66	23.71	
No	18.8	16.29	
**Partner’s educational attainment**			
No education	14.15	11.39	
Primary	19.05	16	
Secondary	25.19	25.19	
Higher	33.03	30.63	
**Religion**			
Hindu	25.08	23.72	
Muslim	23.17	20.13	
Christian	18.93	17.1	
Sikh	30.94	36.84	
Others	32.12	29.88	
**Residence**			
Rural	22.06	18.9	
Urban	32.19	35.52	
**Wealth quintile**			
Poorest	14.26	9.97	
Poorer	19.22	17.23	
Middle	25.54	25.97	
Richer	32.55	32.04	
Richest	36.8	41.07	

*Source* Authors’ calculations

## Model specification and empirical analysis

To assess the impact of the sex of the household head on maternal healthcare utilization, we employ a logistic regression. This is on account of the dependent variable being categorical in nature. Sampling weights applicable for survey data have been incorporated during estimation. The first regression model is as follows:

1 $$mhc{u_i} = \alpha + {\beta _1}{\text{ }}sexhh{d_i} + {\beta _2}{X_i} + {\lambda _s} + {\varepsilon _{\rm I}}$$

Here, mhcu_i_ is the maternal healthcare utilization pertaining to woman i. sexhhd_i_ is the sex of the household head of the woman i. X_i_ is the vector of all individual specific characteristics of the woman, which includes her age, educational attainment level, number of living children, i.e., parity, sex of the last child born, working status, health insurance coverage, exposure to mass media, woman’s participation index in household decision making and possession of an account in a bank or any other financial institution. The results from this estimation have been presented later.

In the second regression model, we further control for partner or spouse-specific characteristics such as partner’s age and educational attainment levels because they are likely to impact maternal healthcare utilization as well. The second regression model is as follows:

2 $$mhc{u_i} = \alpha + {\beta _1}{\text{ sexhh}}{{\text{d}}_{\text{i}}} + {\beta _2}{\text{ }}{X_i} + {\beta _3}{\text{ }}{W_i} + \lambda S + {\varepsilon _i}$$

Where, Wi is the vector for spouse characteristics.

We design another regression model, wherein we control for the household-specific characteristics such as religion, residence, and wealth quintile. The third regression model is thus specified as follows:

3 $$mhc{u_i} = \alpha + {\beta _1}{\text{ sexhh}}{{\text{d}}_{\text{i}}} + {\beta _2}{X_i} + {\beta _3}{W_i} + {\beta _4}{H_i} + \lambda S + {\varepsilon _i}$$

Where, H_i_ controls for household-specific characteristics.

For better model suitability, we add a few more control variables, such as the relationship to the household head and the type of health facility visited, i.e., whether it was a public or private or any other health facility. The last regression model is specified below:

4 $$mhc{u_i} = \alpha + {\beta _1}{\text{ sexhh}}{{\text{d}}_{\text{i}}} + {\beta _2}{X_i} + {\beta _3}{W_i} + {\beta _4}{H_i} + {\beta _5}{B_i} + \lambda S + {\varepsilon _i}$$

Where B_i_ controls for all other additional variables.

The results from all the models are discussed in the following section.

## Results

The results of the regression models are presented in Table 5. We observe that the sex of the household head has a significant influence on maternal healthcare service utilization. The results from model(4) show that women from female-headed households are 19% less likely to use maternal healthcare services fully. This observation is deviant from the general understanding that women would be more concerned with maternal healthcare, and a female household head would thereby influence greater utilization. To explain this dichotomy, we use an interaction term between the sex of the household head and the wealth quintile they belong to. Our results suggest that as female-headed households move up the wealth quintile, the chances of women in those households availing of maternal healthcare services increase.

**Table 5:** Adjusted Odds Ratio from logistic regression

Variables	(Model 1)	(Model 2)	(Model 3)	(Model 4)
Sex of the household head				
Male (Ref)				
Female	0.90^**^	0.91^**^	0.79^**^	0.81^*^
Age	1.03^***^	1.04^***^	1.01^***^	1.01^***^
Educational attainment				
No education (Ref)				
Primary	1.19^***^	1.1	1.01	0.97
Secondary	1.71^***^	1.52^***^	1.17^***^	1.13^**^
Higher	2.19^***^	1.9^***^	1.32^***^	1.25^***^
Parity	0.76^***^	0.77^***^	0.84^***^	0.85^***^
Working status				
Not working (Ref)				
Working	1.04	1.05	1.01	1.01
Health insurance coverage				
No (Ref)				
Yes	1.25^***^	1.25^***^	1.14^***^	1.12^***^
Exposure to mass media				
No exposure (Ref)				
Exposed	1.27^***^	1.25^***^	1.04	1.04
Woman’s Participation Index				
No participation (Ref)				
Participates	1.21^***^	1.21^***^	1.26^***^	1.25^***^
Possession of bank account				
No (Ref)				
Yes	1.24^***^	1.22^***^	1.22^***^	1.18^***^
Sex of the last child				
Male (Ref)				
Female	1.06^*^	1.05^*^	1.06^**^	1.07^**^
Partner’s educational attainment				
No education (Ref)				
Primary		1.22^***^	1.09	1.07
Secondary		1.36^***^	1.14^**^	1.10
Higher		1.38^***^	1.20^**^	1.16^*^
Partner’s age		1.02^***^	1.01^**^	1.01^**^
Religion				
No religion (Ref)				
Hindu			1.04	1.00
Muslim			1.05	1.00
Christian			0.87	0.85
Sikh			1.29	1.23
Others			0.77	0.73
Residence				
Urban (Ref)				
Rural			0.99	0.97
Wealth quintile				
Poorest (Ref)				
Poorer			1.21^***^	1.16^***^
Middle			1.35^***^	1.25^***^
Richer			1.65^***^	1.51^***^
Richest			1.74^***^	1.55^***^
Sex of household head * wealth quintile				
Female* Poorest (Ref)				
Female * Poorer			1.14	1.10
Female * Middle			1.30^*^	1.25
Female * Richer			1.17	1.09
Female * Richest			1.46^**^	1.39^**^
Relationship to the household head				
Head herself (Ref)				
Wife				1.03
Daughter				1.04
Daughter-in-law				1.12
Others				1.23
Type of health facility				
Any other health facility (Ref)				
Public				0.91^**^
Private				1.10^**^
Constant	0.74^***^	0.06^***^	0.11^***^	0.14^***^
State dummies			Included	Included
No. of observations	26,944	26,944	26,944	26,944

^***^p < 0.01, ^**^p < 0.05, ^*^p < 0.1

*Source* Authors’ calculation

The results also show that women in higher age brackets are more likely to utilize maternal healthcare services as compared to younger women. Women in younger age brackets are less likely to be aware of the necessary maternal healthcare seeking and, therefore, end up with inadequate utilization. Women with secondary and higher levels of education are 13% and 25% more likely to use maternal healthcare services, respectively, as compared to women with only primary level of education or no education at all. We further see that the chances of availing maternal healthcare services fall at higher birth orders. Women are 15% less likely to use maternal healthcare services at higher birth orders. Maternal healthcare utilization also increases by 7% if the last-born child is a female. However, the woman’s working status and exposure to mass media do not significantly impact maternal healthcare utilization, as portrayed by our results. We further find that having insurance coverage increases the chances of maternal healthcare utilization by about 12%. Women who possessed a bank account or any account in a financial institution were 18% more likely to utilize maternal healthcare services than those who didn’t hold one. Women who are involved in decision-making instances in their household are 25% more likely to use maternal healthcare services in comparison to women who do not exercise this autonomy.

The likelihood of utilizing maternal healthcare services is 3% lower for women residing in rural areas as compared to their urban counterparts. Moving into a higher wealth quintile increases the odds of access to maternal healthcare services. Women in middle and richer households have 25% and 50% higher chances to gain access to maternal healthcare services, respectively. The results further show that women whose partner has attained higher education have 16% higher chances of utilizing maternal healthcare services. Similarly, the higher the partner’s age, the higher the chances of accessing healthcare services. The relationship to the household head doesn’t impact maternal healthcare utilization, as evidenced by our results. The availability of a private healthcare facility increases the chances of maternal healthcare utilization by 10%.

## Robustness check

In order to check the robustness of the main results, we assess whether they vary across sub-samples. For this purpose, we split the sample based on residence, i.e., rural and urban. The sample size is 21,154 for rural residents and 5790 for urban residents. The results of the regression model specification (Eq. 4) in these sub-sample splits are presented in Table 6. We observe that for the rural sub-sample, women from female-headed households are worse off compared to their male-headed counterparts with regard to maternal healthcare utilization. All the other individual-specific, spouse-specific, household-specific controls and additional controls significantly impact maternal healthcare utilization for the rural sub-sample. The sex of the household head doesn’t substantially impact maternal healthcare utilization in urban areas. We can thereby conclude that the rural areas propel our undivided sample results.

**Table 6:** Robustness check: Sub-sample split based on residence

Variables	Rural (1)	Urban (2)
Sex of the household head		
Male (Ref)		
Female	0.78^*^	2.20
Age	1.02^***^	1.00
Educational attainment		
No education (Ref)		
Primary	0.93	1.23
Secondary	1.15^**^	1.11
Higher	1.32^***^	1.20
Parity	0.82^***^	0.94
Working status		
Not working (Ref)		
Working	0.99	1.07
Health insurance coverage		
No (Ref)		
Yes	1.13^***^	1.10
Exposure to mass media		
No exposure (Ref)		
Exposed	1.03	1.10
Woman’s Participation Index		
No participation (Ref)		
Participates	1.25^***^	1.26^***^
Possession of bank account		
No (Ref)		
Yes	1.16^***^	1.24^**^
Sex of the last child		
Male (Ref)		
Female	1.07^*^	1.07
Partner’s educational attainment		
No education (Ref)		
Primary	1.09	0.95
Secondary	1.10	1.13
Higher	1.18^*^	1.14
Partner’s age	1.00^**^	1.00
Religion		
No religion (Ref)		
Hindu	0.85	1.65
Muslim	0.88	1.54
Christian	0.75	1.36
Sikh	1.09	1.89
Others	0.68	1.00
Wealth quintile		
Poorest (Ref)		
Poorer	1.11^*^	2.00^***^
Middle	1.20^***^	2.00^***^
Richer	1.40^***^	2.67^***^
Richest	1.39^***^	2.97^***^
Sex of household head * wealth quintile		
Female* Poorest (Ref)		
Female * Poorer	1.16	0.38
Female * Middle	1.21	0.58
Female * Richer	1.07	0.43
Female * Richest	1.26	0.57
Relationship to the household head		
Head herself (Ref)		
Wife	1.05	0.99
Daughter	1.08	0.95
Daughter-in-law	1.18	0.98
Others	1.28^*^	1.09
Type of health facility		
Any other health facility (Ref)		
Public	0.87^***^	1.05
Private	1.03	1.35^***^
Constant	0.15^***^	0.07^***^
State dummies	Included	Included
No. of observations	21,154	5790

^***^p < 0.01, ^**^p < 0.05, ^*^p < 0.1

*Source* Authors’ calculation

## Discussion

### Impact of household headship

The results of the study elucidate that women from female-headed households are at a disadvantage when it comes to maternal healthcare service utilization. The interaction term between the sex of the household head and the wealth quintile the household falls into further proves that the utilization of maternal healthcare services increases among female-headed households as they move up the wealth quintile. This holds ground, and previous literature also suggests that female-headed households are poorer than their male-headed counterparts [21–23]. This is because women face occupational segregation and are time-poor because of the unpaid care work burden that leaves them at a disadvantage in the labor market in comparison to men. Therefore, the chances of financial stability are diminished for them. Thus, the income constraint significantly reduces the chances of resource allocation towards healthcare, specifically maternal healthcare in female-headed households. The result is also indicative of the economic vulnerability of female-headed households along with the burden of ever-rising out-of-pocket expenses in private healthcare facilities, which inhibit women from seeking and availing adequate maternal healthcare services.

### Impact of socio-economic factors

In the following section, we briefly discuss the impact of all the other socio-economic variables that we have considered in our study. Older women are more likely to have the necessary knowledge of healthcare utilization, which might increase their chances of healthcare utilization. Similar results have also been found in other studies [6, 7, 13, 16]. Moreover, a higher age at childbirth might increase the chances of obstetric complications and increased maternal and neonatal risks. Such cases of additional risk during pregnancy for older women might call for timely supervision and monitoring, thus leading to their greater utilization of maternal healthcare services. Studies in different developing countries such as Indonesia, Thailand, India, Nepal, and Sub-Saharan Africa have exemplified that education in women enables them to have sufficient knowledge of the benefits of preventive healthcare [6, 8, 13, 17]. Educated women also have improved autonomy in making decisions regarding their own healthcare needs, increasing their likelihood of availing these services [18, 24]. Women who have a basic understanding of reproductive health are better able to communicate with healthcare providers about the problems they face, thereby resulting in quicker detection of problems and provisioning of interventions to save lives. With regard to parity, women are likely to be more cautious and willing to use healthcare services in their first birth, but with subsequent births, they may consider these services redundant, which can be partly a derivation of their previous birth experience or cumbersome healthcare facilities.

We also find that the sex of the last born impacts maternal healthcare utilization. The odds of availing of maternal healthcare facilities are seen to increase if the last child is a female. This likely draws from the fact that the strong son preference in India drives women and their spouses to seek better healthcare in anticipation of a male child. Possession of a bank account or an account in any financial institution implies that those women have some financial literacy or are beneficiaries of government transfer schemes, which, in either way, serves as an instrument to finance their maternal healthcare needs. Furthermore, women’s participation in household decision-making increases their chances of making decisions regarding their own maternal healthcare needs. Women who exercise autonomy have sufficient health literacy to adjudge the benefits of maternal healthcare utilization. A positive relationship between women’s participation in household decision-making and increased utilization of maternal healthcare services is also true in other developing countries such as Ethiopia, Bangladesh, and Indonesia [7, 25, 26].

The likelihood of less service utilization among women in rural areas is due to the lower access that rural women have with respect to healthcare facilities, service providers, proper channels of communication, and transportation, which go a long way in determining healthcare usage. These results are similar to what has been obtained in previous studies [27–30]. This regional disparity leaves rural women at a disadvantage with increased risks of maternal and neonatal mortality. Despite the government provisioning of free maternal healthcare services at public health facilities across India, the increasing tendency towards seeking quality medical care in private health facilities sustains the burden of ever-rising out-of-pocket expenditure [15]. This inhibits households in lower wealth quintiles from utilizing maternal healthcare services. Partner’s age and educational attainment level significantly impact the utilization of maternal healthcare services for women. They are likely to be more aware of pregnancy complications and the benefits of preventive healthcare. This, in turn, increases maternal healthcare utilization. Similar results were also derived in other country contexts [31–35].

## Conclusion

The study aimed at highlighting the influence of the sex of the household head on the utilization of maternal healthcare services. It established that the sex of the household head determines maternal healthcare seeking, and women from female-headed households in India are less likely to utilize maternal healthcare services in comparison to women from male-headed households. This result has been validated in its capacity using an interaction term between the sex of the household head and the household wealth quintile. This goes on to prove from existing studies that the economic vulnerability and reduced income capacity of female-headed households hinder their ability to avail of maternal healthcare services. This study offers a rudimentary insight into the relationship between household headship and maternal healthcare services. The development effort should be inclusive of the gender perspective, especially in the case of sexual and reproductive health. An in-depth understanding of the precarity of maternal health in India has to account for this relationship and consequently devise ways and means that would help marginalized women seek proper maternal healthcare. Women who are uneducated, reside in rural areas, lack an independent income source, and have no exposure to mass media are the most exposed to pregnancy risks and complications. Ensuring maternal healthcare service delivery to them reduces the chances of both infant and maternal mortality. The positive implications of seeking proper maternal healthcare have been highlighted at length. Thus, it becomes imperative for governments and policymakers to design and implement policies that consider these specific maternal healthcare-seeking hindrances and address them adequately.

## Data Availability

The datasets used in this study are publicly available and obtained from the Demographic and Health Surveys. The data can be found here: https://dhsprogram.com/data/dataset/India_Standard-DHS_2020.cfm?flag=0.

## Abbreviations

ANC: Antenatal Care
FHH: Female: Headed Households
MHH: Male: Headed Households
MMR: Maternal Mortality Ratio
PNC: Postnatal Care
SBA: Skilled Birth Assistance
SDG: Sustainable Development Goals
WHO: World Health Organization
